# Chronic Unpredictable Mild Stress in Rats Induces Colonic Inflammation

**DOI:** 10.3389/fphys.2019.01228

**Published:** 2019-09-20

**Authors:** Lina Wei, Ye Li, Wenjun Tang, Qian Sun, Lixin Chen, Xia Wang, Qingyi Liu, Siqi Yu, Shuyan Yu, Chuanyong Liu, Xuelian Ma

**Affiliations:** Department of Physiology, School of Basic Medical Sciences, Shandong University, Jinan, China

**Keywords:** chronic unpredictable mild stress, microbiota, depression, intestinal barrier, colonic inflammation

## Abstract

Chronic psychological stress is associated with an increased risk for relapse of inflammatory bowel diseases (IBD) and impedes the treatment of this condition. However, the impact of stress on the risk of IBD onset remains unclear. The goal of the present study was to examine whether chronic unpredictable mild stress (CUMS) could initiate or aggravate the onset of colon inflammation in rats which, in turn, would be capable of triggering bowel disease. We found that CUMS exposure increased infiltration of CD-45 positive cells and MPO activity, as well as augmented the expression of the inflammatory cytokines, IFN-γ and IL-6 within the colon of these rats. In addition, CUMS treatment changed the composition and diversity of gut microbiota and enhanced intestinal epithelial permeability, indicating the presence of a defect in the intestinal barrier. This CUMS-induced disruption of mucosal barrier integrity was associated with a reduction in expression of the tight junction protein, occludin 1, and an inhibition in mucosal layer functioning via reductions in goblet cells. Results from bacterial cultures revealed an increased presence of bacterial invasion after CUMS treatment as compared with that observed in controls. Thus, our data indicate that CUMS treatment induces alterations of the fecal microbiome and intestinal barrier defects, which facilitates bacterial invasion into colonic mucosa and further exacerbates inflammatory reactions within the colon. Accordingly, chronic stress may predispose patients to gastrointestinal infection and increase the risk of inflammation-related gut diseases.

## Introduction

Chronic psychosocial disorders represent risk factors for the etiology or exacerbation of several gastrointestinal diseases such as functional intestinal disorders and inflammatory bowel diseases (IBDs). For example, it has been reported that persistent stress and stressful life events increase the risk for relapses in patients with ulcerative colitis (UC) (Levenstein et al., 2000; Ringel and Drossman, 2001). Moreover, depression and anxiety are associated with clinical recurrence of IBD (Mikocka-Walus et al., 2016), and patients with depression, as determined at baseline assessment, were at higher risks for worsening of IBD when evaluated at follow-up (Kochar et al., 2018). Overall, IBD patients showed an increased frequency of depression and anxiety as compared to that in the general population, and anti-depressant medications have been shown to improve Crohn’s disease activity indices in some IBD patients (Yanartas et al., 2016). Similar findings were obtained in animal models, as mucosal inflammation was observed within the intestine of rats subjected to chronic stress (Soderholm et al., 2002), and mice exposed to maternal separation stress were not only more susceptible to neonatal intestinal disorders, but also to developing intestinal diseases as adults (Lennon et al., 2013; Murakami et al., 2017). Thus, chronic stress or depression appears to increase the frequency and severity of relapses in IBD patients and impedes the treatment of this disorder. However, the influence of depression or anxiety on the risk of IBD onset remains unclear (Nowakowski et al., 2016). Findings from a retrospective study revealed that some UC patients experienced depression or anxiety at 1 year before the onset of UC, implying that depression may predispose patients to IBD (Kurina et al., 2001). However, whether chronic stress or depression is a risk factor and the mechanisms involved for the onset of developing IBD warrants further investigation.

A number of theories have been presented regarding the means through which depression results in intestinal dysfunction. These include stimulation of the colonic enteric nervous system and secretory motor function, enhanced intestinal permeability, and visceral hypersensitivity (Tache et al., 2017). Recently, a growing number of studies have focused on contribution of the intestinal microbiome as related to the development of gastrointestinal disorders. The gastrointestinal tract is the largest reservoir of commensal bacteria in the human body (Qin et al., 2010; Dimitrov, 2011), harboring at least 100 trillion bacteria which are dominated by four bacterial phyla: Firmicutes, Bacteroidetes, Actinobacteria, and Proteobacteria (Ley et al., 2008). Intestinal microbiota play an important role in maintaining gut health through a variety of mechanisms as several commensal microbes and their metabolites are critically beneficial for the host. Alterations in the composition of the intestinal microbiota, referred to as dysbiosis, contribute to the initiation and maintenance of gastrointestinal diseases. For example, results from several reports have shown that significant reductions in microbial diversity are present in patients with Crohn’s disease and ulcerative colitis (Cotter, 2011; Raoult and Henrissat, 2014; Mangiola et al., 2016). Moreover, attempts to reshape the intestinal microbial ecosystem by probiotic intake or fecal bacteriotherapy exert beneficial effects on chronic intestinal disorders (Makras et al., 2006; Reyes et al., 2010).

It has been suggested that the gut and brain possess a bidirectional communication via the microbiota-gut-brain axis. Recently, increasing evidence has indicated that chronic stress or depression can induce microbial dysbiosis. Chronic water avoidance stress and repeated restraint stress have both been shown to alter bacterial community composition and diversity (Xu et al., 2014), and maternal separation stress in rats results in altered microbiota composition within the pups (Murakami et al., 2017). Interestingly, significant differences in fecal microbiota are present between patients with depression and a control group of non-depressed patients (Naseribafrouei et al., 2014), and attempts to alter gut bacterial communities by rifaximin prevented intestinal inflammation and the impaired permeability induced by chronic stress (Xu et al., 2014). However, the exact mechanisms of these effects remain unclear.

The gastrointestinal barrier is crucial to prevent the entry of pathogenic microorganisms and toxic luminal substances, while allowing efficient transport of nutrients across the epithelium. When breached, microorganisms and toxins within the lumen invade the lamina propria or even the systemic circulation and result in cytokine stress or inflammation of the intestinal mucosa. It has been reported that chronic stress in rats induces enhanced intestinal epithelial permeability (Soderholm et al., 2002; Zheng et al., 2009), and this increase in intestinal permeability has often been related to changes in tight junction proteins (TJ) within the epithelium, such as occludins, junctional adhesion molecule (JAM), and claudins. Although these findings reveal a potential link between psychosocial disease and intestinal disease, the underlying mechanisms are less clear. The goal of this study was to determine whether chronic unpredictable mild stress (CUMS), a reliable procedure for generating a model of depression in rats, could initiate or aggravate the onset of colon inflammation and evaluate some of the possible underlying mechanisms associated with this relationship.

## Materials and Methods

### Animals

Male Wistar rats weighing 180–210 g were purchased from the Shandong University Animal Centre. All animal procedures were approved by the Shandong University Animal Care and Use Committee and were performed in accordance with the National Institutes of Health Guide for the Care and Use of Laboratory Animals. All efforts were made to minimize animal suffering and number of animals used in the experiments. Rats were group housed under SPF conditions and were allowed to adapt to the laboratory conditions for at least 1 week prior to start of the experiments. To avoid the bias that may be introduced by circadian rhythms, all behavioral tests were performed at the same time period of the day (between 09:00 and 11:00 h).

### Experimental Design

**FIGURE 1:**
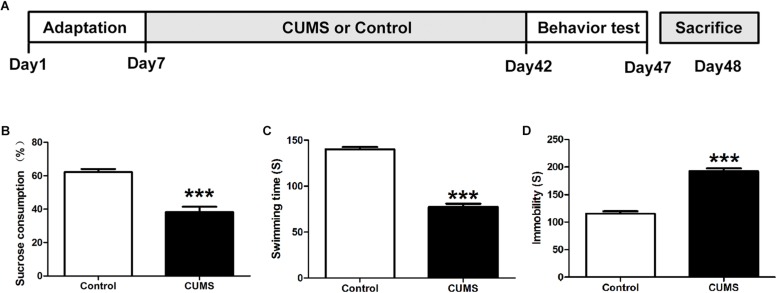
Assessment of depressive-like behaviors. **(A)** Schematic figure of the treatment protocol. **(B)** Five-week chronic unpredictable mild stress (CUMS) treatment decreased consumption of sucrose solution as compared with the control group. **(C)** Five-week CUMS treatment decreased the swimming time**. (D)** Five-week CUMS treatment increased the immobility time. All data are presented as mean ± SEM (*n* = 9). ^∗∗∗^*P* < 0.001 vs. control group.

Rats were randomly assigned to control (*n* = 9) or CUMS (*n* = 9) groups. The CUMS procedure was conducted as previously described (Mao et al., 2009). Briefly, animals were individually placed in acrylic cages for 5 weeks to induce social isolation, at the same time, five cycles of weekly stress regimes were administered, consisting of the following stressors: 45°cage tilt (24 h), food deprivation (48 h), cold swimming (5-min at 4°C), tail pinch (1-min, 1 cm from the distal portion of the tail), physical restraint (2 h), moist bedding (8 h) and overnight stroboscopic lighting. The stressors were administered daily in a random order for five consecutive weeks, with the same stressor not being applied on two consecutive days. Control rats were group housed and were not subjected to any of these stressors throughout the experiment. The following behavioral tests to assess the induction of depression were conducted after the 5-week period of CUMS exposure, after which the rats were euthanized for subsequent assays (Figure 1A).

### Sucrose Preference Test

The sucrose preference test was administered to assess anhedonia as described previously with minor modifications (Liu et al., 2016). In brief, rats were pre-exposed to sucrose in their water for 2 days in the adaptation period. In this period, rats were exposed to two identical bottles containing 1% sucrose solution for 24 h, followed by exposure to two other identical bottles containing either tap water or 1% sucrose solution during the next 24 h period. The rats were then deprived of food and water for another 24 h and the sucrose preference test was then performed on the fourth day of testing. On this day of testing, rats were provided with two identical bottles containing either 100 ml of tap water or 1% sucrose solution and were permitted *ad libitum* access to both bottles for a 3 h period. Sucrose preference was calculated using the following formula: sucrose intake [g]/total fluid intake [g] × 100%.

### Forced Swim Test

The forced swim test was performed to assess despair behavior as described previously (Liu et al., 2016). Rats were individually placed in a cylinder (30 cm diameter, 80 cm height) containing 40 cm of clean water (25°C). The forced swim test was divided into two consecutive sessions: training session and test session. For the training session, rats were initially placed in the cylinder for 15 min to habituate to the testing conditions. On the following day (test session), rats were placed in the cylinder for 5 min and the time spent swimming and immobile was recorded by an observer blinded to the treatment condition of the rats. Swimming time was defined as the sum of all periods of pedaling or making circular movements, while immobility time was defined as the sum of all periods of immobility, that is, when the rat floated with only minimal movements to maintain its head above water. Rats was immediately placed in a dry and clean cage after each swim test. The water in the cylinder was changed after each trial.

After completion of these behavioral tests, the rats were euthanized with pentobarbital sodium (50 mg/kg) and decapitation and samples from the colon were obtained for subsequent assays as described below.

### Histology and Immunohistochemistry Assay

Distal colonic segments were removed, fixed in 4% paraformaldehyde and embedded in paraffin. Sections (4-μm thick), were stained with hematoxylin and eosin (HE) to evaluate any histological damage. In addition, immunohistochemistry assessments of these sections were performed. Briefly, sections were blocked for 1 h at room temperature using blocking buffer (10% normal goat serum, 2.5% BSA, and 0.1% Triton-X-100). Sections were then incubated with primary antibodies overnight at 4°C. Broad Spectrum Zymed Poly HRP conjugated secondary antibodies were used to detected primary antibodies. Color was developed using the ABC reagent. CD45 positive immunoreactivity area percent were manually measured in each field of view using the Image J software.

### Enzyme-Linked Immunosorbent Assay

Cytokines and mucosal activity of myeloperoxidase (MPO) were assessed with use of enzyme-linked immunosorbent assay (ELISA) kits. Briefly, colon samples were homogenized in ice-cold PBS and then centrifuged at 1,000 *g* for 10 min. Supernatants (150 μl) were then collected for MPO assay, with the remainder of the supernatants further centrifuged at 5,000 *g* for 5 min and collected for IL-6, TNF-α, IFN-γ, and IL-10 assay. Cytokines or MPO activity in supernatants were analyzed according to the manufacturers’ instructions. The optical density (O.D.) at 450 nm was measured with use of a microtiter plate reader.

### Ussing Chamber Studies

Ussing chamber studies were performed as described previously (Ma et al., 2018). The proximal colon was removed and placed in oxygenated (95% O_2_–5% CO_2_) Krebs solution at 4°C. Colon segments were sectioned longitudinally along the mesenteric border, washed with ice-cold Krebs solution and serosa and muscular layers removed by blunt microdissection. The resulting mucosal preparations were mounted between the two halves of the Ussing chamber and maintained in oxygenated Krebs solution at 37°C. The membranes were allowed to stabilize for 15 min. Preparations were then voltage clamped to zero and the transepithelial electrical resistance (TEER) was measured.

### Bacterial Culture

A 1-cm portion of the distal colon was harvested. Colon segments were severed, flushed with ice cold sterile PBS, weighed and then homogenized in sterile PBS. Lysates were serially diluted and plated in triplicate on brain heart infusion (BHI) agar plates. After 24 h of incubation at 37°C, bacterial colonies were counted and colony-forming units (CFUs) were normalized to grams of intestinal tissue (CFU/g), representing gut-associated bacterial counts.

### Fecal Microbiota Analysis

Rectal fecal samples were collected after euthanasia and stored at −80°C for 16S rRNA gene sequence analysis. Fecal microbiota DNA was extracted using the CTAB/SDS method according to the manufacturers’ instruction. The V4 variable region of the bacteria 16S rRNA gene was amplified using a pair of universal primers F515 (GTGCCAGCMGCCGCGGTAA) and R806 (GGACTACVSGGGTATCTAAT) with the barcode. PCR products were separated on 2% agarose gel. Only products between 400 and 450 bp were selected and then purified with use of the Qiagen Gel Extraction Kit (Qiagen, Germany). Purified amplicons were sequenced on an IlluminaHiSeq2500 platform and 250 bp paired-end reads were generated. Raw reads were merged using flash and filtered according to the QIIME (V1.7.0) quality control process. Chimeric sequences were identified and discarded using the UCHIME Algorithm. High-quality clean tags were clustered into bacterial operation taxonomic units (OTUs) using Uparse software (Uparse v7.0.1001). Sequences with ≥ 97% similarity were assigned to the same OTUs.

### Western Blotting

Tight junction protein expression was evaluated with use of Western blot. Membrane proteins were extracted using the Mem-PER^TM^ Plus Membrane Protein Extraction Kit according to the manufacturers’ instructions. Distal colon segments (2 cm) were homogenized in ice-cold permeabilization buffer and then centrifuged at 16,000 × *g* for 15 min at 4°C. Following removal of the supernatant, the pellet was resuspended in 1 ml of solubilization buffer and incubated for 30 min at 4°C with constant mixing and then centrifuged at 16,000 × *g* for 15 min at 4°C. The supernatants were then collected for future use. Protein concentrations were determined using BCA reagents. Protein samples were separated on an 8–12% sodium dodecyl SDS PAGE and transferred to a PVDF membrane. The membrane was blocked in 5% non-fat milk with 0.1% Tween-20 and then incubated overnight with specific primary antibodies at 4°C. The membranes were incubated in ECL solution and immuno-detection bands were scanned with use of the Tanon 4600 image system. Band densities were quantified with use of the Image J analyzer software.

### Statistical Analyses

Data are presented as means ± SEM. Statistical comparisons were performed using unpaired *t*-tests with the Prism 6 GraphPad software. A *P*-value less than 0.05 was required for results to be considered as statistically significant.

For gut microbiota analyses, alpha-diversity was evaluated with use of observed species and Shannon index basing on OTUs abundance information using QIIME (Version 1.7.0). A Beta-Diversity index analysis was generated using QIIME software (Version 1.7.0) and expressed by principle co-ordinates analysis (PCoA) and Non-Metric Multi-Dimensional Scaling (NMDS) analysis. The linear discriminant analysis (LDA) effect size (LEfSe) algorithm was used to reveal differences in microbiomes from phylum to species levels between groups. Statistical significance was assessed using R software (Version 2.15.3).

## Results

### Assessment of Depressive-Like Behaviors

Anhedonia and behavioral despair are two core symptoms of depression. In the CUMS animal model of depression used in this report, anhedonia was evaluated with use of the sucrose preference test while behavioral despair by the forced swim test. Compared with the control group, CUMS rats showed significantly less sucrose solution consumption in the sucrose preference test (Figure 1B). With regard to the forced swim test, increased immobility times and decreased swimming times were observed within the CUMS group (Figures 1C,D). These results indicated that CUMS rats were showing behaviors indicative of induced despair/depression.

### CUMS Treatment Induces Colonic Inflammation

**FIGURE 2:**
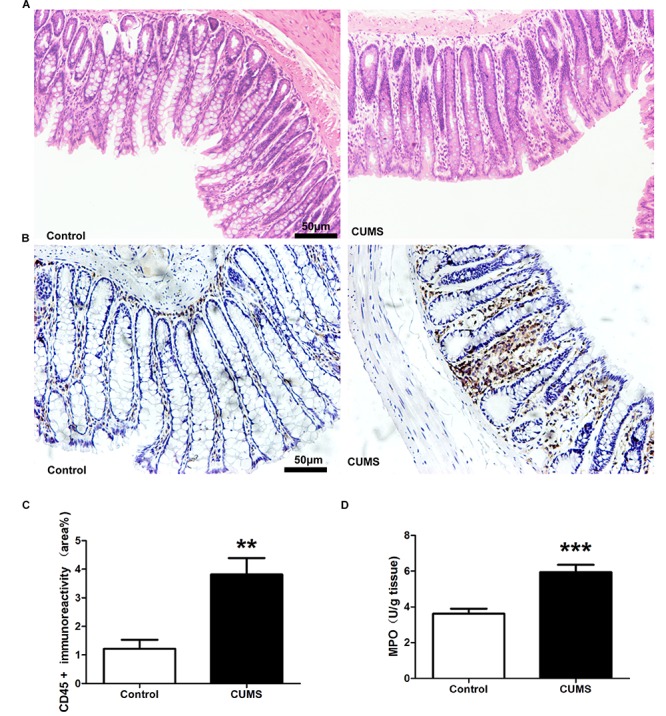
Chronic unpredictable mild stress treatment induces colonic inflammation. **(A)** Representative HE staining showed there were no obvious histological damage. **(B)** Representative immunohistochemical staining for the expression of CD-45. Five-week CUMS treatment significantly increased the CD-45 positive cells infiltration. **(C)** Bar graphs depicting the immunoreactivity of CD-45 positive cells within the different groups. **(D)** Five-week CUMS treatment significantly increased MPO activity within the colon as compared with the control group. All data are presented as mean ± SEM (*n* = 9). ^∗∗^*P* < 0.01 vs. control group; ^∗∗∗^*P* < 0.001 vs. control group.

Hematoxylin and eosin staining was used to assess colonic morphological damages in response to CUMS. Based upon results of this assay no statistically significant differences were observed in colonic damage between the CUMS and control rats (Figure 2A). To investigate the potential role of CUMS treatment upon colonic inflammation, CD-45 immunohistochemisty, and myeloperoxidase (MPO) activity were determined. The results showed that CD-45 positive cells within the colon mucosa were significantly increased in the CUMS treated group as opposed to controls (Figures 2B,C). This CUMS-induced increase in CD-45 was further corroborated by results obtained for MPO, which showed that MPO activity was significantly increased in CUMS rats. These results indicate that increased neutrophil infiltration was present within the colon of rats subjected to CUMS exposure (Figure 2D).

**FIGURE 3:**
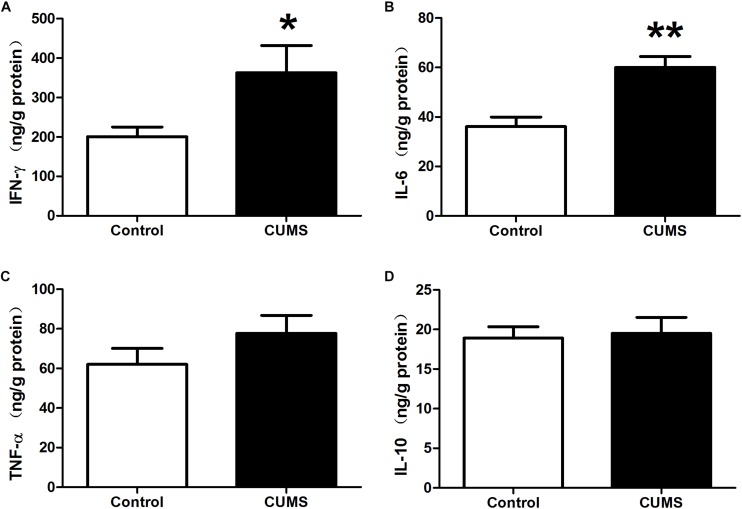
Chronic unpredictable mild stress promotes pro-inflammatory cytokine production in rat colon. **(A)** IFN-γ level within the colon in the different groups as determined using ELISA kits. **(B)** IL-6 level within the colon in the different groups as determined using ELISA kits. **(C)** TNF-α level within the colon in the different groups as determined using ELISA kits. **(D)** IL-10 level within the colon in the different groups as determined using ELISA kits. Values are the mean ± SEM (*n* = 9); ^∗^*P* < 0.05 vs. control group; ^∗∗^*P* < 0.01 vs. control group.

Enzyme-linked immunosorbent assay results demonstrated that IFN-γ and IL-6 levels within the colon of the CUMS group were significantly increased as compared with those in the control group (Figures 3A,B). Although TNF-α levels were also higher in the CUMS group, this increase failed to achieve statistical significance (Figure 3C). Nor were there statistically significant differences between CUMS and control rats in IL-10 levels (Figure 3D).

### CUMS Treatment Induces Alterations of the Fecal Microbiome

**FIGURE 4:**
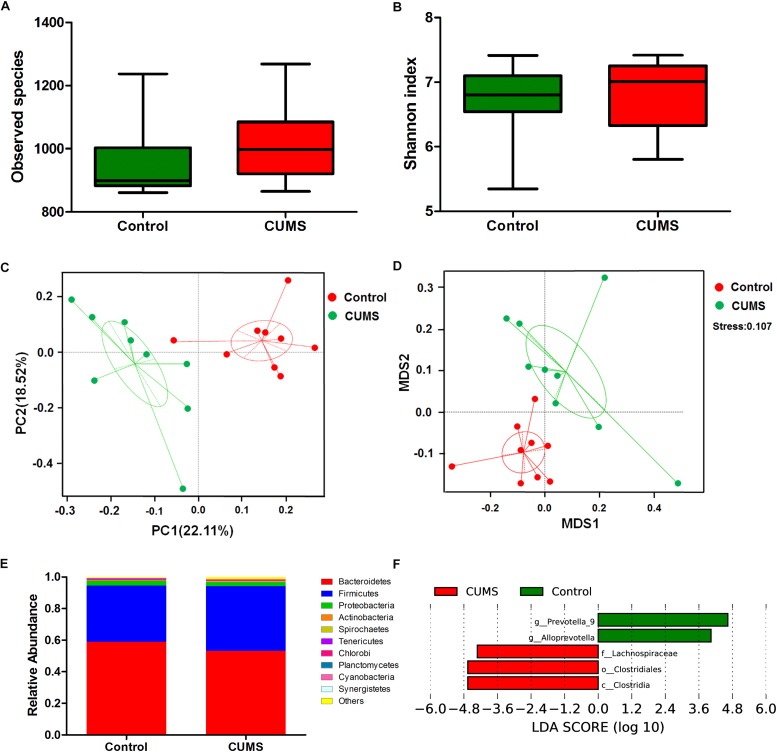
Chronic unpredictable mild stress treatment induces microbial composition changes in mouse rectal fecal samples. **(A)** The effect of 5-week CUMS treatment on the observed species. **(B)** The effect of 5-week CUMS treatment on Shannon index. **(C)** Principle coordinate analysis (PCoA) plot between the CUMS and control group. There was an obvious separation between the control and CUMS group along the first principal component (PC1) axis. **(D)** Non-Metric Multi-Dimensional Scaling (NMDS) plot showed obvious separation between the control and CUMS group, the stress is 0.107. **(E)** Taxonomy comparisons at phylum level show the 10 most abundant phyla in different groups. **(F)** Bacterial taxa with significantly different abundances between the control and CUMS groups, as determined by linear discriminant analysis (LDA) effect size (LEfSe) analysis. The bar charts report the taxonomic representation of statistically and biologically consistent differences between the two groups.

Alpha diversity (within-community diversity) analysis including observed species and Shannon index were evaluated. The results showed that no statistically significant difference was found between the CUMS group and the non-stressed control group, indicating that the within-community diversity was not significantly altered after CUMS treatment (Figures 4A,B).

Beta diversity analysis was evaluated through PCoA and NMDS to assess differences in microbial composition between the two groups. Ordination of Bray–Curtis dissimilarity by PCoA revealed an obvious separation between the control and CUMS groups along the first principal component (PC1) axis (Figure 4C). Consistently, the NMDS plot revealed the same pattern, which substantiated the results obtained as described above, and the stress value of 0.107 indicated that these differences were statistically significant (Figure 4D).

Taxonomy comparisons at the phylum level for both groups were performed and the relative abundance is shown in Figure 4E. Dominant phyla in the control group were Bacteroidetes (58.96%), Firmicutes (35.48%), Proteobacteria (2.98%) and Actinobacteria (0.68%), while those in the CUMS group were Bacteroidetes (53.25%), Firmicutes (40.9%), Proteobacteria (2.43%), and Actinobacteria (0.33%). These results demonstrate that CUMS treatment leads to a decrease of Actinobacteria. To further analyze the main microbes altered in response to CUMS treatment, the LDA effect size (LEfSe) algorithm was used to compare bacteria taxa from phylum to species between the CUMS and control groups. As shown in Figure 4F, three bacterial taxa were enhanced after CUMS treatment, including the family *Lachnospiraceae*, Order *clostridiales* and Class *clostridia*, all of which belong to the phylum Firmicutes. Conversely, the genera *prevotella-9* and *Alloprevotella* were significantly reduced by CUMS treatment, both of which belong to the phylum Bacteroidetes. Although Actinobacteria at the phylum level decreased after CUMS treatment, the difference between the CUMS and control groups were not significant. And there were no bacterial clades which belong to the phylum Actinobacteria were significantly changed when detected using LEfSe algorithm.

### CUMS Treatment Increased the Intestinal Permeability and Bacterial Penetration Within the Colonic Mucosal Layer

**FIGURE 5:**
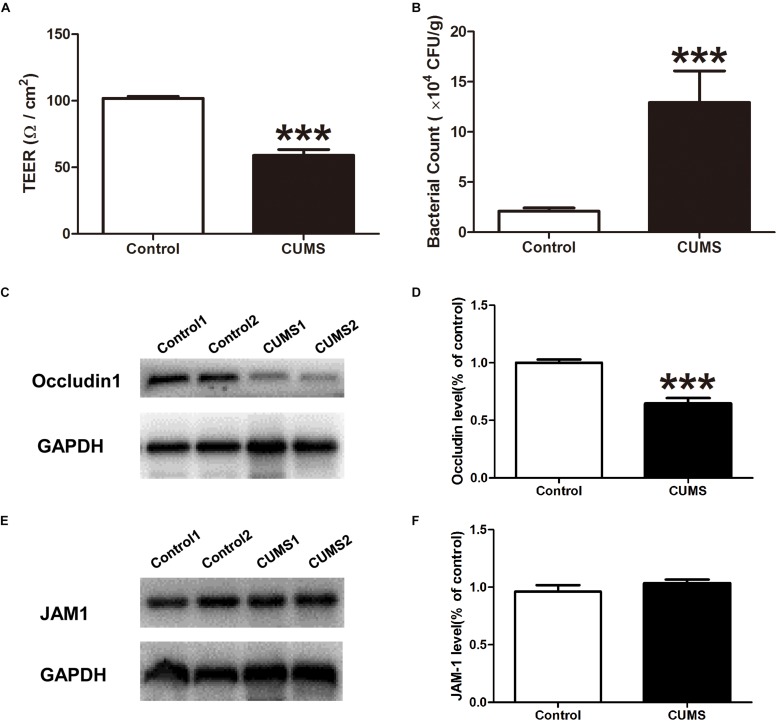
Chronic unpredictable mild stress treatment triggers intestinal barrier defects. **(A)** TEER was measured within the different groups with use of the Ussing chamber. **(B)** Bacterial counts in colonic tissues were determined with a colony-forming assay using BHI agar. **(C)** Representative western blot analysis of the expression of occludin1 in mice colon mucosa in normal and CUMS group. **(D)** The bar graph shows relative protein levels. Mean of occludin1 protein ratios to GAPDH in the control group is expressed as 1. **(E)** Representative western blot analysis of the expression of JAM1 in mice colon mucosa in normal and CUMS group. **(F)** The bar graph shows relative protein levels. Mean of JAM1 protein ratios to GAPDH in the control group is expressed as 1. All data are presented as mean ± SEM (*n* = 9). ^∗∗∗^*P* < 0.001 vs. control group.

Ussing chamber studies were used to evaluate epithelial barrier integrity. The TEER of the colon mucosa in the control group was 101.8 ± 1.43 Ω/cm^2^ while that in the CUMS group was significantly reduced to 59.0 ± 4.3 Ω/cm^2^ (Figure 5A). In addition, when assessing the presence of bacteria within colon mucosa, we found that CUMS treatment significantly increased bacterial penetration within colon mucosa as compared to controls, as demonstrated in BHI agar (Figure 5B).

### CUMS Treatment Inhibited Occludin 1 Expression in Colon Epithelia

The expression of occludin 1 and JAM 1 proteins in colon epithelia were evaluated with use of western blot analysis. As shown in Figures 5C,D, exposure to CUMS significantly suppressed the expression of occludin 1, but not that of JAM1 protein expression (Figures 5E,F).

### CUMS Treatment Decreased Goblet Cell Number and Mucus Production Within the Colon

**FIGURE 6:**
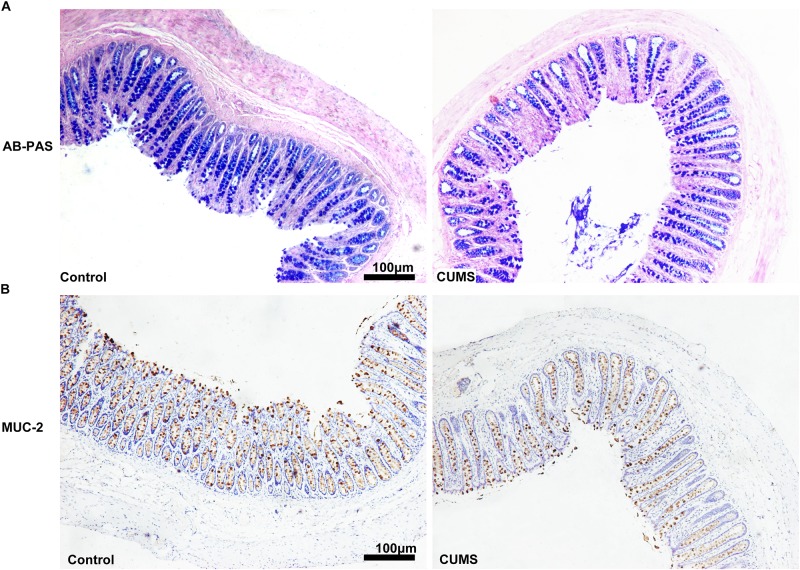
Chronic unpredictable mild stress treatment decreased goblet cell numbers and mucus production in colon. **(A)** Five-week CUMS treatment dramatically decreased the mucus production**. (B)** Five-week CUMS treatment dramatically decreased the goblet cell numbers.

To evaluate mucous layers, AB-PAS staining was used. As shown in Figure 6A, the mucous layer was remarkably reduced after CUMS treatment as compared with that in control group. In addition, CUMS exposure also decreased the goblet cell number within the colon mucosa as determined by evaluating muc-2 expression (Figure 6B).

## Discussion

While emerging evidence has recently revealed that depression exerts negative effects on the course, flare-up rates and treatment outcomes of IBD, the influence of these psychological disorders on the risk of IBD onset remains unclear (Kochar et al., 2018). In the current study, we provide evidence that CUMS treatment can induce colonic inflammation in rats, indicating a potential role for depression in the initiation of colitis. Furthermore, our results demonstrate that CUMS-induced colonic inflammation is, at least in part, induced by alterations of the intestinal microbiome and breakdown of the intestinal mucosal barrier. Such effects can, in turn, contribute to bacterial penetration into the colonic mucosa and thus lead to inappropriate immune responses and inflammation within the colon.

To evaluate the validity of our CUMS model of depression, the sucrose preference and forced swim tests were used. Results from the sucrose preference test showed that after 5-weeks of CUMS exposure, rats showed significantly reduced consumption of the sucrose solutions. These responses indicate anhedonia, which is one of the main symptoms of depression. Similarly, in the forced swim test, CUMS-exposed rats showed significantly decreased swim time and significantly increased immobility time as compared with non-stressed control rats. These responses indicate despair or helplessness. Taken together, these behavior results demonstrate that our 5-week CUMS procedure lead to depression-like behavior in rats.

Soderholm et al. (2002) first reported that chronic psychological stress can induce primary gut inflammation in a previously healthy host. Consistent with that report, our results showed that 5-weeks of CUMS exposure induced an extensive infiltration of CD45-positive leukocytes within rat colon and enhanced the infiltration of activated neutrophils as measured by MPO. However, despite this remarkable degree of cellular infiltration within colonic mucosa, there was no evidence of histological damage in these colons. Results from ELISA assays demonstrated that increased expressions of pro-inflammatory factors, such as TNF-α and IL-6, were present in colonic tissue. Accordingly, these findings indicate that although no obvious histological damage was induced, CUMS treatment did, in fact, initiate colonic inflammation in these CUMS rats.

The role of gut microbiota has recently received increasing attention with regard to their contribution to human health, in particular, to that involving intestinal and psychiatric disorders. As an approach to assess this eventuality, we examined the potential for CUMS exposure to induce changes of the fecal microbiome. To accomplish this goal, 16S rRNA gene sequence analysis was used to analyze the bacterial community composition and diversity. In accord with previous studies, our results showed that the CUMS exposure substantially altered the structure of gut microbiota. While it has been reported that healthy subjects harbor relatively greater levels of Bacteroidetes within the gut (Shen et al., 2017), in the majority of IBS patients, Bacteroidetes were decreased, along with increased Firmicutes at phylum levels (Jeffery et al., 2012). We observed a similar shift with Bacteroidetes of 58.96% in the control group decreasing to 53.25% in the CUMS group, while Firmicutes at phylum levels increased to 40.9% in the CUMS group from 35.48% in the control group (Figure 4E). These results provide new information on the main bacterial taxa that are altered in response to CUMS exposure. The findings that CUMS exposure significantly reduced the genera *prevotella-9* and *Alloprevotella*, would contribute to the observed decrement of the phylum *Bacteroidetes.* It is well-known that *Prevotella* can produce short chain fatty acids (SCFAs), which play important roles in a number of processes involved with maintenance of gut health (Fernández et al., 2016). For example, SCFAs serve as the main energy source of colonocytes (Parada Venegas et al., 2019) and promote epithelial barrier function (Kelly et al., 2015). In addition, SCFAs induce transcription of the junction protein, Claudin-1, protect intestinal barrier function (Wang et al., 2012) as well as suppress colon inflammation by inhibiting the colonization of opportunistic pathogens in the gut (Chassard and Bernalier-Donadille, 2006). In this way, SCFAs reduce the growth of *Salmonella*, which is a common foodborne pathogen (El-Gedaily et al., 1997). Recent findings have further shown that SCFAs also exert immunomodulatory functions within the intestine (van der Beek et al., 2017). Therefore, a decrease of *Prevotella* may, at least in part, induce an increment of mucosal permeability and mucosal inflammation. In the present study, SCFA concentration hasn’t been detected, additional work is needed in the future to elucidate if the fecal SCFA is reduced because of the decreased *Prevotella*. Our current results show that the Class *clostridia*, Order *clostridiales and* family *Lachnospiraceae* were increased following CUMS administration. These data are consistent with a previous report showing that gut *clostridia and Lachnospiraceae* were increased in patients with major depressive disorder (Cheung et al., 2019). In a related report, increased levels of intestinal *clostridia* were correlated with impaired differentiation of IL-10-secreting regulatory T lymphocytes *in vitro*, an effect which contributed to an inhibition of immunoregulatory functions in multiple sclerosis (Cekanaviciute et al., 2018). On the basis of these observations we postulate that CUMS treatment induces alterations of the fecal microbiome, facilitates the outgrowth of *clostridia and Lachnospiraceae* and inhibits the growth of *Prevotella*, all of which may then contribute to the induction of colitis. Consistent with our observations, is the work by Tian et al. (2019) who demonstrated that microbiota dysbiosis was induced by chronic stress. However, studies by Huang et al. (2018, 2019) showed that Actinobateria were increased and Firmicutes were decreased in humans with major depressive disorder. The inconsistency of these studies may be due to the difference of experimental models and treatment. And further studies are needed in the future.

We then explored some of the potential mechanisms of bacterial invasion and colonic inflammation resulting from CUMS treatment. We focused on the intestinal barrier, as an impairment in this structure represents an essential aspect of IBD pathogenesis. Results from numerous studies have demonstrated that stress increases gut permeability through activation of the hypothalamic pituitary adrenal (HPA) axis and/or local activation of mast cells within the intestine (Soderholm et al., 2002; Farhadi et al., 2007; Wallon et al., 2008; Moussaoui et al., 2016). Moreover, commensal bacteria are vital for functional integrity of the epithelial barrier (Rakoff-Nahoum et al., 2004) while dysbiosis, resulting from stressful conditions, facilitates the induction of intestinal hyperpermeability (Collins and Bercik, 2009). In line with these previously reported studies, we found that the TEER in rat colonic mucosa was significantly reduced after 5-weeks of CUMS treatment, implying that a primary intestinal epithelial barrier defect was induced. The destruction of tight junctions represents a major factor contributing to epithelial dysfunction, therefore we directed our attention to examining the expression of the TJs, occludin1 and JAM1, in rat colonic epithelia, as these have been reported to play central roles in the physical regulation of paracellular permeability (Van Itallie and Anderson, 2006). Our results demonstrated that CUMS treatment significantly reduced the expression of occludin 1, but had no effect on JAM1 protein expression. Accordingly, these findings showing that CUMS treatment inhibits the expression of barrier-forming components of TJs, suggest that the decrease of occludin 1 expression in colon mucosa may be partly responsible for the “leaky” epithelial barrier. As the intestinal mucous layer is also a very important component of the intestinal barrier, we further assessed the effects of CUMS upon mucous layers and goblet cell numbers within the colon of these rats. We found that both the mucous layer and goblet cell numbers were significantly reduced after CUMS exposure. These results indicate that reductions in the mucous layer and number of goblet cells may also contribute to intestinal hyperpermeability.

It is important to specify limitations associated with this study. Overall, the complex interplay that exists among CUMS, intestinal microbiota and mucosal inflammation, makes it difficult to establish a clear cause-effect relationship among these factors. Moreover, while colonic inflammation could be due to CUMS-induced alterations of the fecal microbiome, a reverse causality is also possible. Accordingly, while our results provide some important new data concerning IBD as induced by depression or anxiety, further studies will be required to clarify additional biological mechanisms involved in this relationship.

## Conclusion

The results of the present study demonstrate that 5-weeks of CUMS treatment alters the fecal microbiome structure and community, thus leading to increases in opportunistic pathogens and a decrease in some beneficial bacteria necessary for maintaining intestinal health. Moreover, CUMS treatment led to intestinal barrier defects and intestinal hyperpermeability, effects which in turn enable the invasion of opportunistic pathogens into colonic tissue. The combined effects of these alterations induced by CUMS may then be responsible for the colonic inflammation observed. Overall, our findings confirm that chronic ongoing psychological stress can induce alterations of the fecal microbiome, dysfunction of mucosal barrier and finally result in intestinal inflammation in healthy rats. Such results provide strong evidence indicating that chronic stress may predispose patients to gastrointestinal infection and increase the risk of inflammation-related gut diseases.

## Data Availability Statement

The datasets generated for this study are available on request to the corresponding author.

## Ethics Statement

The animal study was reviewed and approved by Shandong University Animal Care and Use Committee.

## Author Contributions

XM and ShY designed the study and wrote the manuscript. YL, LW, WT, and QS performed the experiments. XW provided technical support. LC, QL, and SiY performed the statistical analysis. CL helped analyze data and provided scientific advice.

## Conflict of Interest

The authors declare that the research was conducted in the absence of any commercial or financial relationships that could be construed as a potential conflict of interest.
